# Proteomic profiling reveals treatment-dependent inflammatory signatures and identifies biomarkers of refractory age-related macular degeneration

**DOI:** 10.3389/fphar.2026.1793714

**Published:** 2026-06-16

**Authors:** Chiara M. Eandi, Angelica Borgo, Eva de Oliveira Figueiredo, Diego de Haro, Daniel E. Kaufmann, Raphael Roduit

**Affiliations:** 1 Department of Ophthalmology, University of Lausanne, Jules-Gonin Eye Hospital, Lausanne, Switzerland; 2 Department of Surgical Sciences, University of Torino, Turin, Italy; 3 Division of Infectious Diseases, Department of Medicine, Lausanne University Hospital (CHUV) and Université de Lausanne, Lausanne, Switzerland

**Keywords:** age-related macular degeneration (AMD), angiogenic factors, anti-VEGF drugs, inflammation, multiplexing, olink-PEA, refractory AMD (rAMD)

## Abstract

**Background:**

Age-related macular degeneration (AMD) is a severe eye disease that affects the macula, the central area of the retina. Current treatment consists of intravitreal injections of vascular endothelial growth factor (VEGF) inhibitors. However, some patients remain refractory to treatment even after maximal anti-VEGF dosage. We longitudinally analyzed angiogenic and inflammatory biomarkers in the aqueous humor (AH) of these patients.

**Objectives:**

1) To confirm the presence of angiogenic and inflammatory biomarkers before (in the naive state) and after two anti-VEGF injections; and 2) to characterize the inflammatory response according to the type of anti-VEGF used (ranibizumab, aflibercept, brolucizumab, or faricimab).

**Methods:**

AH samples were collected from two groups of patients: 35 patients with AMD and 17 control patients with no retinopathy. Eleven of the 35 patients with AMD were characterized as refractory (rAMD) after long-term anti-VEGF treatment. Multiplex and proximity extension assays (PEA) were performed on these samples to define their inflammatory profiles. Free VEGFA was detected using an additional AlphaLISA VEGFA assay.

**Results:**

Several inflammatory molecules are upregulated in AMD, while a smaller subset is downregulated in rAMD, indicating that these could serve as markers of a refractory state. In contrast, no differences were observed in the biomarkers previously reported as increased in rAMD after long term treatment. These findings suggest that anti-VEGF treatment induces the expression of angiogenic and inflammatory biomarkers. Analysis of the inflammatory response following the injection of different anti-VEGF agents revealed exacerbated inflammation in patients treated with brolucizumab, whereas ranibizumab caused the lowest inflammatory response. In addition, we compared three different assays to assess AH VEGFA, but only the AlphaLISA assay could detect free VEGFA.

**Conclusion:**

Our results indicate that anti-VEGFA therapy shapes AMD-associated inflammation; therefore, the choice of anti-VEGFA therapy remains important in reducing secondary inflammatory effects. Furthermore, the detection of free VEGF is dependent on the assay used.

## 1 Introduction

Age-related macular degeneration (AMD) is a severe ocular disease affecting the macula, which is the central area of the retina. It is a leading cause of irreversible vision loss in individuals older than 50 or 55 years of age in developed countries. AMD is classified into two subgroups: the dry (geographic atrophy) and wet (exudative) forms. The wet form is linked to choroidal neovascularization (CNV) directed to the subretinal macular region, which may result in a loss of central vision. Currently, no AMD cure is available at disease onset. The only medical treatment offered is for the CNV, and it consists of anti-vascular endothelial growth factor (VEGF) therapies, including ranibizumab (Brown et al., 2006; Rosenfeld et al., 2006), aflibercept (Heier et al., 2012), brolucizumab (Holz et al., 2016), or faricimab (Khanani et al., 2020). Although these treatments are the gold standard, a considerable portion of patients never achieve a complete response despite receiving the maximum anti-VEGF treatment every month (Fogli et al., 2018). These patients are often referred to as having refractory AMD (rAMD); they exhibit pathologic intraretinal or subretinal fluid on optical coherence tomography (OCT) despite monthly treatment. The reasons for this refractoriness remain unclear (Yang et al., 2016).

We recently studied rAMD using a proteomic approach. Patients with AMD characterized by incomplete anti-VEGF response displayed an increased inflammatory response, complement activation, cytolysis, protein-lipid complex, and vasculature development pathways (Mantel et al., 2020). We subsequently identified several biomarkers in patients with rAMD at late follow-up timepoints. However, it remains unclear whether these proteins are induced during anti-VEGF treatment or are already present at higher levels in the aqueous humor (AH) of patients with future rAMD at baseline (before treatment initiation). In the present study, we aimed to analyze AH at a naïve state, with a follow-up of 2 months (after two anti-VEGF injections). For clarity, when we talk about patients whose analyses were performed before their health status (refractory or good responder) was determined, we refer to future refractories or responders. In addition, we sought to evaluate the inflammatory response induced by different anti-VEGF drugs. Various studies (Cox et al., 2021) and our clinical observations suggest that the inflammatory response may differ depending on the anti-VEGF agent used.

## 2 Materials and methods

We performed this prospective pilot study at the Jules-Gonin Eye Hospital, Medical Retina Unit, and the fundamental research laboratory of the same institution, which are grouped within the Foundation Asile des Aveugles (Lausanne, Switzerland). The study protocol was approved by the local ethics committee (CER-VD; protocol ID 340-15). This was conducted in accordance with all national legal requirements and the tenets of the Declaration of Helsinki. All participants provided written informed consent.

### 2.1 Samples collection

The study used AH from two groups of participants: patients diagnosed with AMD and treated with different anti-VEGF drugs (AMD group) and control patients with no retinal pathologies that underwent cataract surgery (control group). AH was collected from naïve patients with AMD before treatment. We subsequently followed up with the same patients after 2 months and two anti-VEGF injections, including ranibizumab (7 patients), aflibercept (10 patients), faricimab (13 patients), and brolucizumab (5 patients) (Table 1). Refractoriness to anti-VEGF treatment was defined as the presence of persistent intra- (IRF) or subretinal fluid (SRF) on spectral-domain OCT after three to six consecutive monthly anti-VEGF injections, despite confirmed anatomical and functional stabilization attempts (Supplementary Table S2). The refractoriness was established at the 6th month of treatment, while tested samples were used after 1-2 injections. In the control group, AH was collected at the beginning of cataract surgery, immediately after initial paracentesis The AH was stored in the biobank until further analyses.

**TABLE 1:** Demographic and treatment characteristics of each group based on the type of molecular analysis.

	AMD				Ctrl
*N* patients (H/F)	35 (11/24)				17 (6/11)
*N* patients: (anti-VEGFA: Rani./Afli./Brolu./Fari.) (H/F)	1/6	6/4	2/3	10/3	—
Mean age ± SEM	80.6 ± 1.1				70.8 ± 2.3
Mean age ± SEM (anti-VEGFA: Rani./Afli./Brolu./Fari.)	78.5 ± 1.8	83.7 ± 1.7	82.2 ± 2.7	79.3 ± 2.1	—
Rigth eyes/Left eyes	12/23				8/9
Refractory AMD (%) – (H/F)	11 (31.4%) – (1/10)				—
Mean age ± SEM (Refractory vs Responder)	82.1 ± 1.4		80.3 ± 1.5		—
Neovascularization type (1/2/3/PCV)	15/11/7/2				—
Presence of reticular pseudodrusen (%)	14 (40%)				—
Presence of fibrosis (%)	6 (17.4%)				—
Presence of cRORA (%)	6 (17.4%)				—
Presence of iRORA (%)	7 (20%)				—
Récurrence including IRF (%)	19 (54.3%)				—
Récurrence including SRF (%)	28 (80%)				—
Mean CRT in micrometers (SEM)	370.8 (±12.8)				—

The anti-VEGF drugs used are ranibizumab (Rani), aflibercept (Afli), faricimab (Fari), and brolucizumab (Brolu), polypoidal choroidal vasculopathy (PCV).

The exclusion criteria for both groups were as follows: any confounding retinopathy; diabetes (independent of presence or absence of diabetic retinopathy); insufficient visibility of the fundus for retinal diagnosis; any anterior segment eye surgery in the study eye within 3 months preceding AH collection; any posterior segment surgery within 6 months; any preceding treatment such as photodynamic treatment, laser, or intraocular or periocular steroids within the 6 months preceding AH collection; and inability to provide informed consent.

AH samples from the biobank were selected by the laboratory investigator (R.R.), whereas no further clinical data corresponding to the coded data from the biobank were available to the laboratory investigator. In contrast, the clinical investigator (C. E.) had full access to the patient files and imaging documentation. All files corresponding to the coded AH samples were validated by the clinical investigator according to the inclusion and exclusion criteria. In addition to the attributes of the study group, the following clinical data were included in the coded study database: age; sex; and anti-VEGF treatment agent (ranibizumab, aflibercept, faricimab, or brolucizumab).

Samples were analyzed by multiplex and proximity extension (PEA) assays. Additional measurements for VEGFA detection were performed by AlphaLISA assay.

### 2.2 Samples analysis

#### 2.2.1 Multiplex analysis

Multiplex analysis was performed using Luminex xMAP technology (ProcartaPlex; Thermo Fisher Scientific, Waltham, MA, USA) as previously described (Mantel et al., 2020). This enabled the inclusion of many human cytokines, chemokines, growth factors, and other protein targets (Supplementary Figure S1A). Target proteins were selected based on our (Mantel et al., 2020) and other previously published studies that showed their presence in AH (Liu et al., 2016; Jonas et al., 2012; Sato et al., 2018) or serum (Nassar et al., 2015) from patients with AMD, or based on studies that showed the involvement of these proteins in AMD pathology (Dias et al., 2011; Knickelbein et al., 2015). We detected 19 of the 25 analytes using 10 µL of AH in duplicate according to the manufacturer’s instructions. Owing to the limited AH volumes available and the use of some samples for PEA inflammatory measurements, the final number of test results varied among analytes. Not all samples were tested with both the multiplex assay and PEA.

#### 2.2.2 PEA for inflammatory measurements

AH samples were collected according to best standard practice. A final volume of 1 µL per sample was analyzed using the Olink Target 96 Inflammation panel, a high-sensitivity multiplex immunoassay based on PEA technology (Supplementary Figure S1B). This method enables the simultaneous quantification of 92 protein markers associated with inflammation and disease pathways. Briefly, paired antibodies conjugated with unique oligonucleotide tags bind to their target protein overnight. When in proximity, these tags hybridize and are then extended by standard PCR to create a unique amplicon for each marker. Finally, these amplicons are loaded onto a microfluidic chip and quantified using qPCR in the Olink Q100 machine. Experiment quality control (QC) is assessed on the NPX Signature software (v.2). In this study, all assays and samples passed internal and external QC metrics, except for one sample (timepoint 2 months post-Faricimab injection in Patient 8) that was subsequently excluded. The limit of detection (LOD) for each assay is defined as the mean NPX value of the three negative controls plus three standard deviations (mean +3 SD). The negative control samples consist of buffer to assess background signal generated when antibody pairs come into random proximity and produce a DNA tag. In total, 43/92 proteins were above the LOD. As this study is a small exploratory studies, we keep below-LOD values, as excluding them might risk losing real biological signal and discovery, particularly when the data follow a normal distribution. This is especially relevant for assays with values close to the LOD or for comparisons where one group predominantly falls below the LOD while the other is above it. Normalized protein expression (NPX) values on a log2 scale generated by the NPX Signature software were used without correction for age, sex, or other clinical parameters in downstream statistical analysis.

#### 2.2.3 AlphaLISA assay

A homogenous no-wash immunoassay (AlphaLISA; PerkinElmer, Waltham, MA, USA) was used to measure VEGFA and compare results obtained with the multiplex and PEA analyses. We used 2 μL (in triplicate) of AH to perform the assay. The AlphaLISA assay contained acceptor beads coated with an anti-VEGF antibody, streptavidin-coated donor beads, a biotinylated anti-VEGFA antibody, a lyophilizate VEGFA (for the standard curve), and an assay buffer (10×). After incubation with all components, the signal was quantified using an EnVision multimode plate reader (PerkinElmer). Competition experiments with anti-VEGF drugs, including ranibizumab, aflibercept, bevacizumab, and brolucizumab, were performed in the presence of 30 nM VEGFA and an increasing amount of each drug, ranging from 10 to 10,000 ng/mL.

### 2.3 Statistical analysis

**FIGURE 1:**
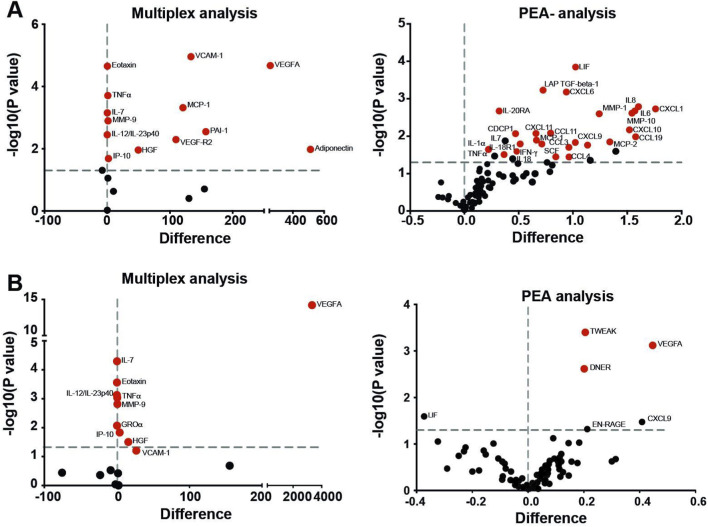
Multiplex and PEA analysis of AH. **(A)** Graphic representation of molecules detected in the AH of treatment-naïve patients with AMD and controls. **(B)** Graphic representation of molecules detected in the AH of treatment-naïve and treated patients with AMD. Statistical analyses were conducted using multiple-paired and unpaired t-tests with Welch correction, with statistical significance set at p < 0.05. Volcano plots showed raw p-values, while red color highlight significant biomarkers analyzed in multiple comparison analysis with Benjamini-Hochberg FDR at 10%.

**FIGURE 2:**
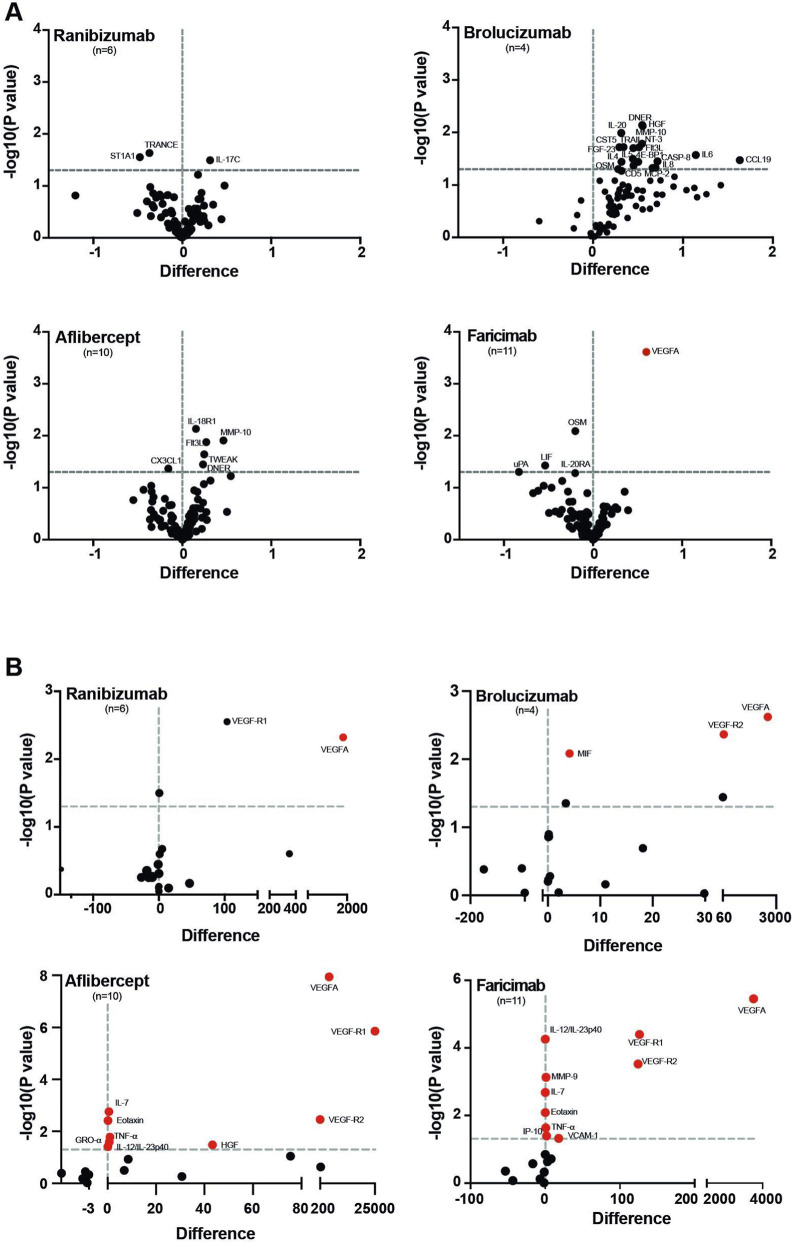
Multiplex and PEA analysis of AH categorizing patients based on the drug they received Vulcano plots representation of molecules detected by PEA **(A)** and by multiplex **(B)** in AMD patients treated with the specific anti-VEGF drugs, including ranibizumab (6), aflibercept (10), faricimab (11), and brolucizumab (4). Statistical analyses were conducted using multiple-paired and unpaired t-tests with Welch correction with statistical significance set at p < 0.05. Volcano plots showed raw p-values, while red color highlight significant biomarkers analyzed in multiple comparison analysis with Benjamini-Hochberg FDR at 10%.

**FIGURE 3:**
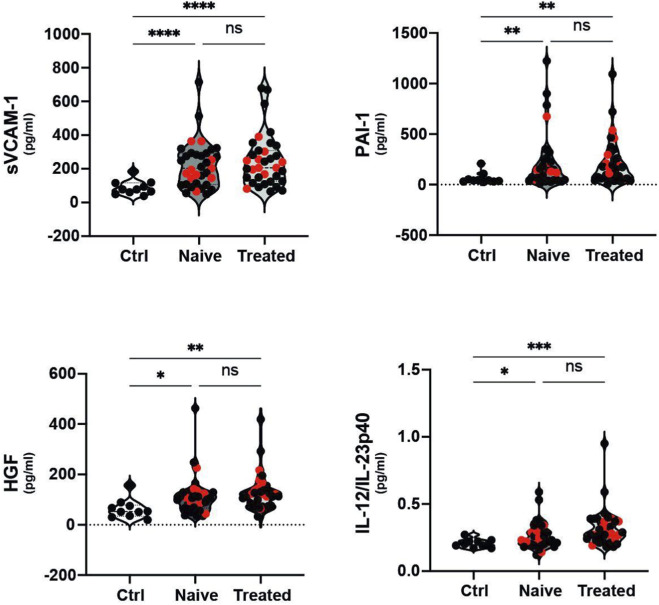
Multiplex analysis of AH from control (Ctrl), naïve nAMD (Naïve), and treated nAMD (Treated) patients. Graphic representation of sVCAM-1, PAI-1, HFG, and IL-12p40. Results are expressed as the mean ± SEM, as determined using the Brown-Forsythe and Welch Anova tests (*p < 0.05, **p < 0.005, and ****p < 0.0001). Refractory patients with AMD (rAMD) are shown in red.

Multiple paired and unpaired t-tests with Welch correction and corresponding volcano plots, as well as the multiple comparisons analyses using Benjamini-Hochberg FDR at 10%, were conducted using GraphPad Prism (v.10.4.1); statistical significance was set at p < 0.05. Pearson correlation (p < 0.05) was used to analyze the correlation between the multiplex assay and PEA. Vulcano plots (Figures 1–3) report raw p-values, while red color highlight significant biomarkers analyzed in multiple comparison with corrected values. Both raw and corrected values are available in supplemental excel files (PEA-Sumarize_V2.xlsx and Multiplex-Sumarize_V2.xlsx).

## 3 Results

The clinical characteristics of all patients are summarized in Table 1; AH was used in multiplex, PEA, and AlphaLISA analyses. This table also shows ranibizumab, aflibercept, brolucizumab, and faricimab distribution, the mean age of each treated group and gender repartition among treated patients with AMD.

### 3.1 Naïve AMD vs. control patients

We first analyzed the AH of naïve patients with AMD in comparison with that of control patients not affected by AMD, using both multiplex assays and PEA (Figure 1A). As expected, we detected many molecules that have been previously described to be involved in AMD. Significantly higher concentrations of VEGFA were detected by multiplex analysis in patients with AMD, along with many other cytokines, including soluble vascular cell adhesion molecule-1 (sVCAM-1), hepatocyte growth factor (HGF), plasminogen activator inhibitor type 1 (PAI-1), tumor necrosis factor alpha (TNFα), adiponectin, eotaxin, monocyte chemoattractant protein-1 (MCP-1), matrix metalloproteinase (MMP)-9, interleukin (IL)-7, 12/23p40 (IL12/23p40), and CXCL10 (also known as interferon gamma-induced protein 10, IP-10) (Supplementary Material for p value and difference of expression). Using the PEA, we also detected several other inflammatory molecules (Supplementary Material for p value and difference of expression) in addition to those detected by multiplex analysis. Taken together, these results confirm the key role of inflammation in triggering neovascularization in AMD.

### 3.2 Naïve patients with AMD vs. those treated for 2 months

We compared the AH of naïve patients with AMD to that collected after 2 months of monthly anti-VEGF treatment (Figure 1B). Multiplex analysis detected an increase in the levels of several cytokines (IL-12/IL-23p40, IL-7, TNFα, eotaxin, IP-10, MMP-9, growth-regulated oncogene alpha (Groα), and HGF), while PEA analysis detected an increase of TWEAK and Delta/notch-like epidermal growth factor (EGF)-related receptor (DNER). Both assays showed a significant increase of VEGFA. To elucidate the effects of various anti-VEGF drugs on inflammation, we categorized participants based on the drug they were administered (Figure 2). We observed a trend of increase in IL-17C levels among ranibizumab-treated patients, whereas the levels of sulfotransferase family 1 A member 1 (ST1A1) and TNF-related activation-induced cytokines (TRANCE) decreased. The trend of expression of more molecules was upregulated in aflibercept-treated patients, including that of IL-18R1, MMP-10, TNF weakly inducer of apoptosis (TWEAK), DNER, Fms-related tyrosine kinase 3 ligand (Flt3L), and chemokine [C-X3-C motif] ligand 1 (CX3CL1). These diverse signaling proteins (cytokine receptors, enzymes, and ligands) are involved in immune responses, tissue remodeling, cell survival, and inflammation. Patients treated with faricimab showed no increase in the expression of tested inflammatory molecules; they exhibited a trend in the decrease in the expression of leukemia inhibitory factor (LIF), oncostatin M (OSM), interleukin-20 receptor subunit α (IL-20RA) and urokinase-type plasminogen activator (uPA). In contrast, brolucizumab-treated patients showed a trend in the increase in the expression of numerous inflammatory molecules following two injections. These included IL-4, -5, -6, -8 and -20; DNER; HGF; MMP-10; neurotrophin 3 (NT3); fibroblast growth factor 23 (FGF-23); Flt3L; caspase 8 (CASP8); C-C motif chemokine ligand 19 (CCL19); OSM; CD5; eukaryotic translation initiation factor 4E binding protein 1 (4EBP-1); tumor-necrosis-factor related apoptosis inducing ligand (TRAIL), and monocyte chemotactic protein 2 (MCP-2). These results confirm our clinical observations, showing that inflammation is more pronounced after brolucizumab injection than with other anti-VEGF treatments (Supplementary Material for p value and difference in expression). Although the objective of anti-VEGF treatment is to reduce active VEGFA, we observed an increase in VEGFA levels with all anti-VEGFA drugs used. This unexpected result will be explained later in the manuscript in relation to the anti-VEGFA assay that was used. Similar analyses were conducted using the multiplex assay (Figure 2B), categorizing patients based on the drug they received. Interestingly, excepted for a significant increase of VEGFA in all four groups, the levels of certain molecules are significantly higher in patients treated with aflibercept and faricimab, including IL-7, eotaxin, TNF-α and IL-12/IL-23-p40. Brolucizumab treated patients also showed a significant increase of macrophage inhibitory factor (MIF).

### 3.3 Biomarkers in patients with rAMD

We previously showed an increase in sVCAM, PAI-1, HGF, IL12/23p40, and IL-6 levels in the AH of patients that were refractory to the anti-VEGF after long-term treatment (Mantel et al., 2020). We hypothesized that these markers either contribute to the lack of response to anti-VEGF treatment or are induced by it. Among the 35 patients whose data were analyzed, we identified 11 patients who became refractory with time after multiple anti-VEGF injections, regardless of the anti-VEGF molecule used (Table 1; Supplementary Table S1). These biomarkers were significantly increased in the AH of naïve patients with AMD compared to those in control non-affected patients (Figure 3). However, no clear difference was detectable between future refractory patients (in red in the graph) and future good responders to the treatment. Nevertheless, none of the biomarkers previously identified in the AH of patients with rAMD after long-term treatment were significantly increased in naïve patients or in those who had received two treatments for AMD. IL-6 was barely detectable in these AH. Therefore, this result is difficult to analyze. Taken together, these results could suggest that the previously described biomarkers in long-term refractory patients could likely reflect the early stages of subsequent resistance or could be due to a treatment-related modulation.

**FIGURE 4:**
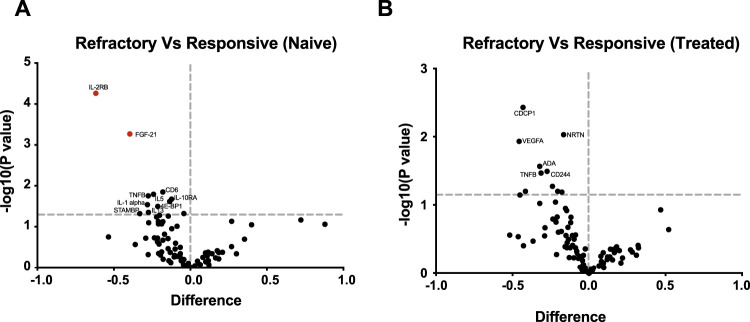
PEA analysis of AH from control (Ctrl), naïve nAMD (Naïve), and treated nAMD (Treated) patients. **(A)** Graphic representation of molecules detected in the AH of rAMD (Naïve) and responsive patients. **(B)** Graphic representation of molecules detected in the AH of rAMD (Treated) and treated patients with AMD. Statistical analyses were conducted using multiple paired and unpaired t-tests with Welch correction and corresponding volcano plots, with statistical significance set at p < 0.05. Molecules with a significant difference are annotated and shown in red.

The levels of several inflammatory molecules were significantly decreased in future AMD refractory participants compared with those in future AMD responders. The expression of several inflammatory molecules decreased significantly in both treatment-naïve patients (Figure 4A; Supplementary Figure S1A) and those who had received two anti-VEGF injections (Figure 4B; Supplementary Figure S1B).

### 3.4 VEGFA measurement by different assays

**FIGURE 5:**
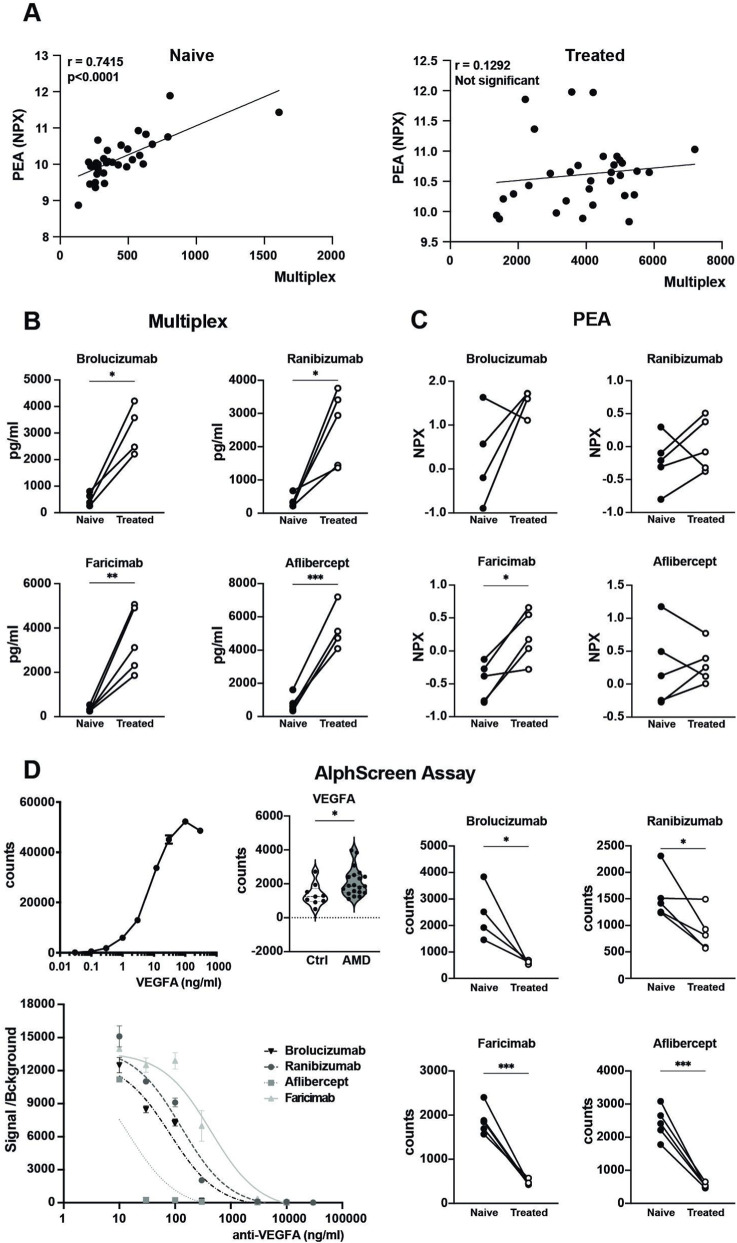
Measurement of VEGFA in the AH of naïve (Naïve) and treated (Treated) patients by different assays. **(A)** Graphic correlation between both multiplex assay and PEA in naïve **(a)** and treated **(b)** patients. Graphic representation of VEGFA levels in patients treated with four different anti-VEGF agents (brolucizumab, ranibizumab, faricimab, or aflibercept) (n = 4 patients per group), as measured by multiplex **(B)**, PEA **(C)**, and AlphaLISA **(D)** assays. Results are expressed as the mean ± SEM, as determined using a paired t-test (*p < 0.05, **p < 0.008, and ****p < 0.0008).

**FIGURE 6:**
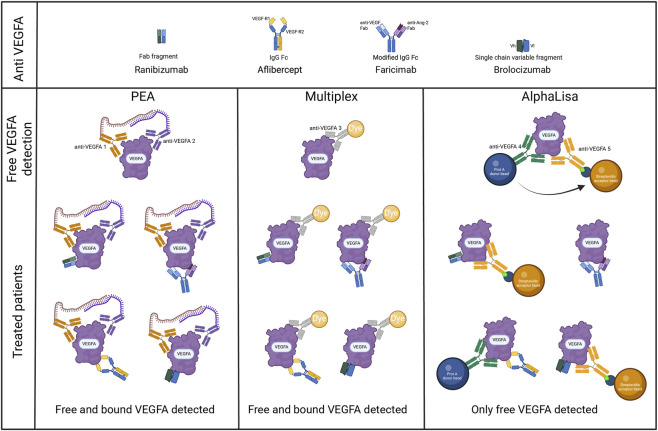
Schematic illustration of the potential interactions of each anti-VEGF drug, based on the assays used. Anti-VEGFA, including ranibizumab, aflibercept, faricimab and brolocizumab are described and anti-VEGFA (1-5) are specific antibodies used in each different assays (created with BioRender.com).

VEGFA expression is typically increased in patients with AMD, and this was observed in multiplex assays and PEA (Figure 1A). In contrast, the concentration of VEGFA should be decreased following anti-VEGF treatment. However, increased levels of VEGFA were observed in the AH of treated patients (Figure 1B) when measuring by multiplex and PEA. A positive correlation between the two techniques was observed when analyzing samples from naive patients, but no correlation was observed when analyzing those from treated patients (Figure 5A). This could be explained by the fact that both assays measure total VEGFA (free VEGFA and the complex VEGFA/anti-VEGF). To test this hypothesis, we analyzed results of four identical AH samples obtained by multiplex assays and PEA and compared them with those obtained through AlphaLISA. Multiplex analysis revealed a significant increase in VEGFA concentrations in treated patients compared to that in naïve patients; this result was obtained in all four treatment groups (Figure 5B). However, PEA yielded different results (Figure 5C). We observed a significant increase in VEGFA concentrations among faricimab-treated patients, whereas the results were less consistent in the other three patient groups. We first tested VEGFA–AlphaLISA by evaluating the dose response of VEGFA (Figure 5D), that showed a low level of detection (10 ng/mL) and a high linearity of 3 log. Comparison of VEGFA levels in the AH of AMD and control patients showed an expected significant increase in the angiogenic factor. The same samples (tested by both multiplex and PEA assays) were then tested by AlphaLISA, and a significant decrease was observed in all treated patients (Figure 5D). We further performed a competitive assay with an increase in the concentration of anti-VEGF drugs and a stable amount of VEGFA (Figure 5D). All drugs decreased the signals, with EC50 comparable to that in published data (nanomolar range). These results strongly suggest that AlphaLISA measures free VEGFA, whereas multiplex assays and PEA measure total VEGFA. Figure 6 hypothetically explain these results. Indeed, antibodies used for the detection of VEGFA are different depending of the assay. The results suggest that the antibodies recognize different epitopes to those targeted by anti-VEGF drugs. In this case, both bound and free VEGF are detected. The opposite is true for the AlphaLisa assay, where the antibodies recognize the same antigenic regions as anti-VEGF drugs. It is also possible that these antibodies have a lower affinity, meaning VEGF predominantly binds to anti-VEGF drugs rather than to the antibodies used in the AlphaLisa assay. This leads to the detection of free VEGF.

## 4 Discussion

Our study had two objectives: to identify specific biomarkers in patients who are refractory to anti-VEGF treatment and verify the inflammatory response in patients depending on the anti-VEGF used. We showed that both multiplex analyses and the recent PEA techniques were suitable approaches. However, PEA was more advantageous; 1 µL of AH was sufficient to obtain highly accurate and reliable data, detecting approximately 93 molecules.

In our previous study, the angiogenic process and inflammatory response were involved in patient refractoriness to anti-VEGF treatment (Mantel et al., 2020). Consistent with these findings, multiplex analysis highlighted an increase in the expression of sVCAM-1, IL-6, IL-12p40, PAI-1, and HGF among patients with rAMD, compared to non-AMD controls, in the present study. Therefore, we analyzed the AH of patients who became refractory. These analyses were first conducted before treatment (naïve state) to determine whether these molecules were already highly expressed in these patients, consequently increasing the likelihood of refractoriness. We subsequently analyzed the AH of the same patients after two injections of anti-VEGF, thereby monitoring each patient. The expression of these biomarkers was increased in both naïve and treated patients with AMD. However, they were not differentially expressed in AMD patients who have received two injections but who will become refractory after long-term treatment compared to treatment responders. These results suggest that the activated angiogenic and inflammatory pathways previously detected in rAMD after long-term treatment are due to anti-VEGF treatment. Analysis of inflammatory pathways using PEA revealed a decrease in the expression of many molecules (Figure 3). FGF-21, which was significantly decreased in both naïve and treated patients with rAMD, was previously linked to AMD. In particular, FGF-21-KO mice were more susceptible to AMD in a previous study, suggesting that insufficient levels of this strong anti-inflammatory metabolic regulator could be prognostic for refractoriness and may be a therapeutic target for AMD (Zhao et al., 2022), further investigation needs to be done. IL-2RB levels were also significantly decreased in rAMD. However, no direct link has been established with AMD or rAMD to date. IL-2RB deficiency leads to immune system dysfunction (Zhang et al., 2019). Similarly, CD244 regulates immune responses (Agresta et al., 2018), but to our knowledge has never been linked to AMD. The decrease in IL-10RA and IL-10RB levels, which are receptors for the anti-inflammatory IL-10 that facilitate the inhibition of IL-6, IL-8, and TNFα (Verma et al., 2016), likely leads to reduced inhibition and exacerbated inflammation. GDNF, a neurotrophic factor involved in the survival and function of retinal neurons (Baranov et al., 2017), here also reduce in baseline rAMD, is a potential target for AMD therapy. The decrease in TNFB and IL-1α expression is more difficult to understand because both molecules are normally increased in AMD. Thus, we expected a similar increase in rAMD. We further compared the levels of the molecules with decreased expression among 2 months-treated patients (rAMD versus AMD good responders). We also observed a decrease in the levels of various markers among patients with rAMD compared to those in good responders. Although CDCP1 may play a role in the integrity of the retinal pigmented epithelial barrier (Zhang et al., 2022), it seems to be negatively corelated with T-cell infiltration and inflammation. Neurturin, a member of the GDNF super family of ligands, is implicated in neurodegenerative diseases. However, no links with AMD or rAMD have been established to our knowledge.

The involvement of inflammation in AMD has long been recognized (Borchert et al., 2023). Analysis of AH using PEA in the present study successfully answered our second question: whether the inflammatory response differs depending on the anti-VEGF used. In this study, we identified inflammatory proteins involved in AMD and induced by anti-VEGF treatment (Figures 1A,B). Numerous studies highlight inflammatory pathways in AMD (Tan et al., 2020), and our results (Figure 1A) confirm the implications of these pathways in this pathology. A recent bidirectional two-sample Mendelian randomization study showed that elevated levels of four molecules—C–C motif chemokine ligand 11 (CCL11), signaling lymphocytic activation molecule family member 1 (SLAMF1), TRANCE, and VEGFA—have a positive impact on AMD (Wang et al., 2024). Our results were consistent with this study for CCL11 and VEGFA (significantly increased in AMD) but did not confirm any modification in the expression of the other two molecules (Figure 1A). Considerably different results were observed when we separately analyzed the effect of each anti-VEGF drugs on inflammatory molecules. A few inflammatory molecules were induced in ranibizumab-treated patients, whereas higher levels of inflammatory cytokines were observed in aflibercept-treated patients. These results are consistent with the higher incidence of intraocular inflammation reported for brolucizumab, both in clinical trials and in real-world studies (approximately 4%–10%), compared with that reported for other anti-VEGF agents (generally <3%). Our findings demonstrate significantly elevated inflammatory cytokine levels in the AH of patients treated with brolucizumab, supporting a heightened intraocular inflammatory response associated with this molecule (Anderson et al., 2021; Cox et al., 2021; Patil et al., 2022; Park et al., 2024). Interestingly, pro-inflammatory cytokines such as MIF are high in these patients and have been described as central mediators of the inflammatory process (Zhang et al., 2023; Rivera et al., 2024).

VEGFA levels were elevated in patients with AMD at baseline, supporting anti-VEGF therapy as the established treatment approach using various anti-VEGF agents. Accordingly, accurate assessment of VEGFA is a key element in evaluating treatment effects. Although both multiplex and PEA analyses confirmed increased VEGFA levels in treatment-naïve patients with AMD (Figure 1A), the results obtained after anti-VEGF therapy were unexpected (Figure 1B). These findings echo the concerns raised by Wakshull et al. regarding the importance of assay selection in relation to the specific experimental question (Wakshull et al., 2018). Comparison of free VEGFA levels among patients treated with different anti-VEGF agents is challenging, if not impossible, due to drug-specific interactions with VEGFA. Consistent with this hypothesis, we observed markedly different VEGFA levels when measured using the VEGFA AlphaLISA assay, which employs antibodies distinct from those used in the multiplex assay and PEA. As shown in Figure 5C, AlphaLISA demonstrated a three-log linear detection range and confirmed significantly elevated VEGFA levels in patients with AMD. However, VEGFA was undetectable by AlphaLISA in patients treated with brolucizumab, ranibizumab, faricimab, or aflibercept, indicating complete signal disruption by these agents. These data suggest that AlphaLISA specifically measures free VEGFA, whereas multiplex and PEA assays likely detect VEGFA–anti-VEGF complexes (Figure 6). In other words, anti-VEGFA antibodies used in each assay may have an impact on the measure of free VEGFA.

In accordance with the recommendations of the PEA developers and common practice in many publications (Sun et al., 2023), we decided to retain values below the limit of detection (LOD). However, there is a risk that the magnitude of the effect may be distorted within the non-linear range. To address this issue, we have explicitly indicated which markers had a mean value above or below the LOD (see Supplementary Figures S1, S2) and have highlighted the results that are potentially affected (e.g. IL-2RB, see Supplementary Figure S1). Limitations of this study include the relatively small number of patients in each group, which is even more true for the group of patients treated with brolucizumab (n = 4). Despite this low number, a significant increase in the expression of many inflammatory proteins was observed. Same observation could be done for future refractory patients which have different neovascularization types; this heterogeneity makes it difficult to include them in the statistical analysis given the small number of cases for each type of lesion. Owing to the availability of a new anti-VEGF molecule and increasing clinical evidence of severe intraocular inflammation, we limited the use of brolucizumab as a third line drug to reduce this risk in our patients, leading to a lower number of AH samples in this group. The control group was not completely age-matched with the AMD groups and also showed a mild imbalance in sex distribution. However, the molecules with significant differences between the groups are not known to be age- or sex-dependent. In addition, part of this study focused on the differences in the inflammatory process under different anti-VEGF treatments, which were matched according to age and gender. As this is a pilot study, further analyses involving a larger sample size will be necessary to validate these results.

## 5 Conclusion

Multiplex analyses did not reveal increased levels of sVCAM, PAI-1, HGF, IL-6, or IL-12p40 in patients with AMD showing an incomplete response to anti-VEGF therapy, either at the treatment-naïve stage or after treatment. These findings suggest that the elevations of these biomarkers previously observed after long-term anti-VEGF exposure are more likely early trajectories associated with subsequent refractoriness rather than intrinsic to incomplete therapeutic response. In contrast, PEA analyses revealed a significant reduction in several inflammatory markers in the AH of patients with refractory AMD, providing new insights into the inflammatory profile associated with incomplete response. However, the causal relationship between these molecular changes and persistent retinal fluid requires confirmation in larger, longitudinal studies. Nonetheless, our data highlight the potential of high-sensitivity proteomic approaches to refine disease stratification and identify novel therapeutic targets aimed at improving outcomes in neovascular AMD, such as FGF21 (potentially prognostic biomarker for refractoriness) and other biomarkers. Importantly, inflammatory profiling using the Olink-PEA platform further revealed that brolucizumab is associated with a more pronounced inflammatory signature than other anti-VEGF agents, which is coherent with current literature and clinical practice. This underlines the value of molecular monitoring to support timely treatment adaptation and enhance patient safety. Our results strongly confirm that the inflammatory response to the treatment depends on the type of anti-VEGF agent used. Due to the small size of the cohort, this trend needs to be validated in a larger AMD cohort.

## Data Availability

The original contributions presented in the study are included in the article/Supplementary Material, further inquiries can be directed to the corresponding author/s.
