# Supplementary material for: Proteomic profiling reveals treatment-dependent inflammatory signatures and identifies biomarkers of refractory age-related macular degeneration
Source: Front Pharmacol. 2026 Jun 16;17:1793714. doi: 10.3389/fphar.2026.1793714 (PMC13315084; doi:10.3389/fphar.2026.1793714)

Supplementary Material

# Supplementary Data

Supplementary Data S1 (Multiplex-Summarize.xlsx) summarize all results obtained with multiplex assay, while Supplementary Data S2 (PEA-Summarize.xlsx) summarize results obtained with PEA assay. Each tab, of the excel file, describes the samples and the type of anti-VEGFA used.

# Supplementary Figures and Tables

## Supplementary Tables

**A**

| Adiponectin | EGF | Eotaxin | Eotaxin-3 | GRO alpha | Ghrelin |
| --- | --- | --- | --- | --- | --- |
| IL-12/IL-23p40 | IFN gamma | IL-1 beta | HGF | IL-1RA | IL-3 |
| IL-6 | IL-7 | IP-10 | MCP-1 | MCP-3 | MIF |
| MMP-9 | PAI-1 | TNF alpha | VCAM-1 | VEGF-A | VEGF-R1 |
| VEGF-R2 |  |  |  |  |  |

**B**

| 4E-BP1 | CD5 | FGF-21 | IL18 | LIF-R | STAMBP |
| --- | --- | --- | --- | --- | --- |
| ADA | CD6 | FGF-23 | IL-18R1 | MCP-1 | TGF-alpha |
| ARTN | CD8A | FGF-5 | IL2 | MCP-2 | TNF |
| AXIN1 | CDCP1 | Flt3L | IL-20 | MCP-3 | TNFB |
| Beta-NGF | CSF-1 | GDNF | IL-20RA | MCP-4 | TNFRSF9 |
| CASP-8 | CST5 | HGF | IL-22 RA1 | MMP-1 | TNFSF14 |
| CCL11 | CX3CL1 | IFN-gamma | IL-24 | MMP-10 | TRAIL |
| CCL19 | CXCL1 | IL-1 alpha | IL-2RB | NRTN | TRANCE |
| CCL20 | CXCL10 | IL10 | IL33 | NT-3 | TSLP |
| CCL23 | CXCL11 | IL-10RA | IL4 | OPG | TWEAK |
| CCL25 | CXCL5 | IL-10RB | IL5 | OSM | uPA |
| CCL28 | CXCL6 | IL-12B | IL6 | PD-L1 | VEGFA |
| CCL3 | CXCL9 | IL-13 | IL7 | SCF | CCL4 |
| DNER | IL-15RA | IL-8 | SIRT2 | CD244 | EN-RAGE |
| IL-17A | LAP TGF beta1 | SLAMF1 | CD40 | FGF-19 | IL-17C |
| LIF | STA1A1 |  |  |  |  |

**Supplemental Table 1:** **Multiplex and PEA assays**. (A) Cytokines present on the ProcartaFlex and (B) Targets 96 Inflammation panel present in PEA assay

**
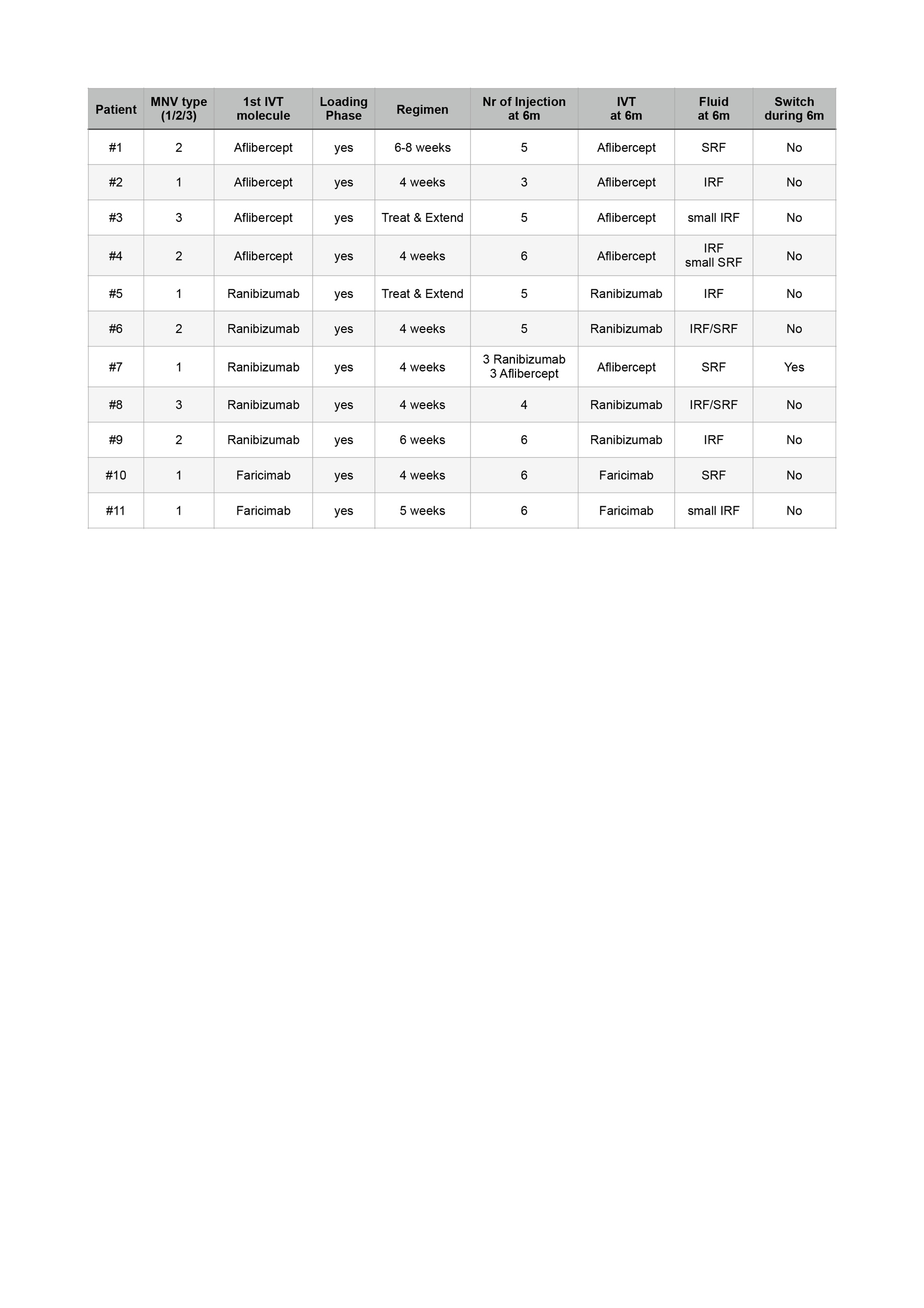
**

**Supplemental Table 2: Characteristics of refractory patients**. MNV= membrane neovascular; IVT= intravitreal injection; IRF= intraretinal fluid; SRF= subretinal fluid

## Supplementary Figures


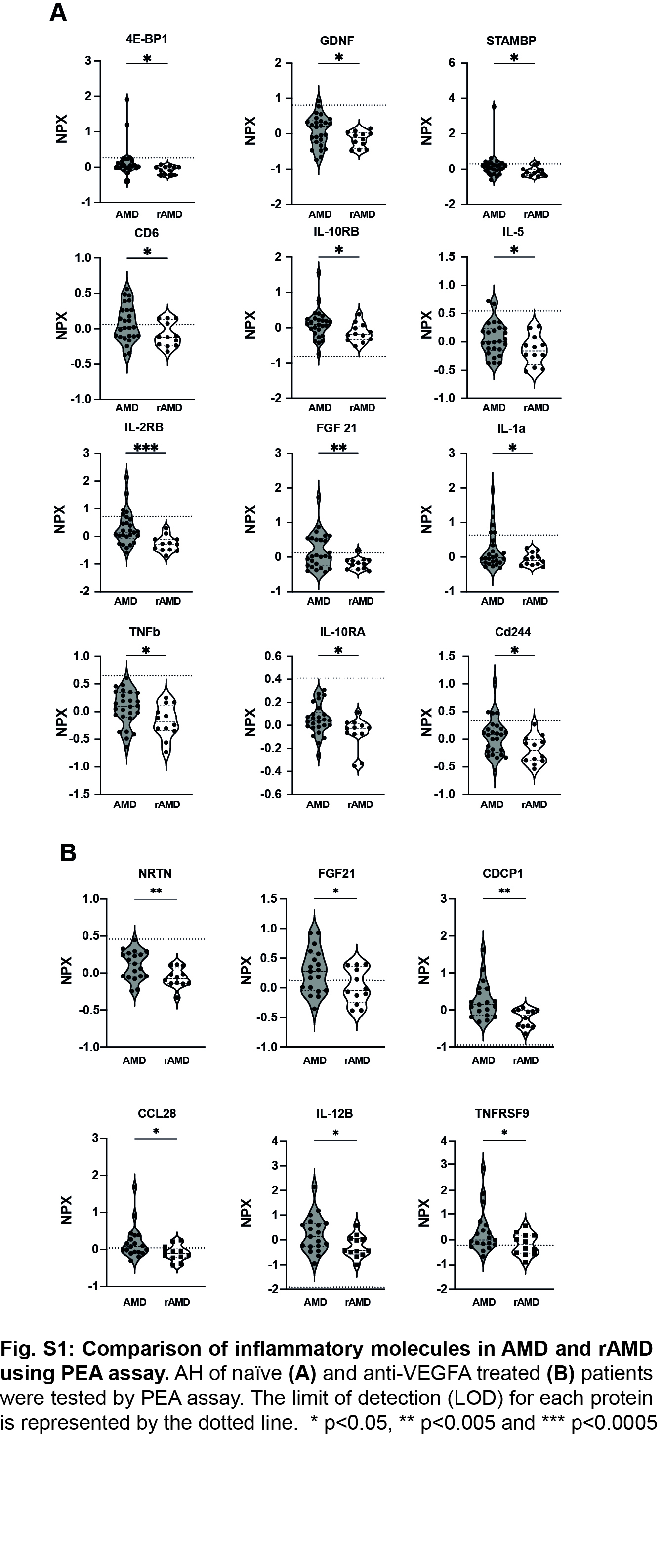


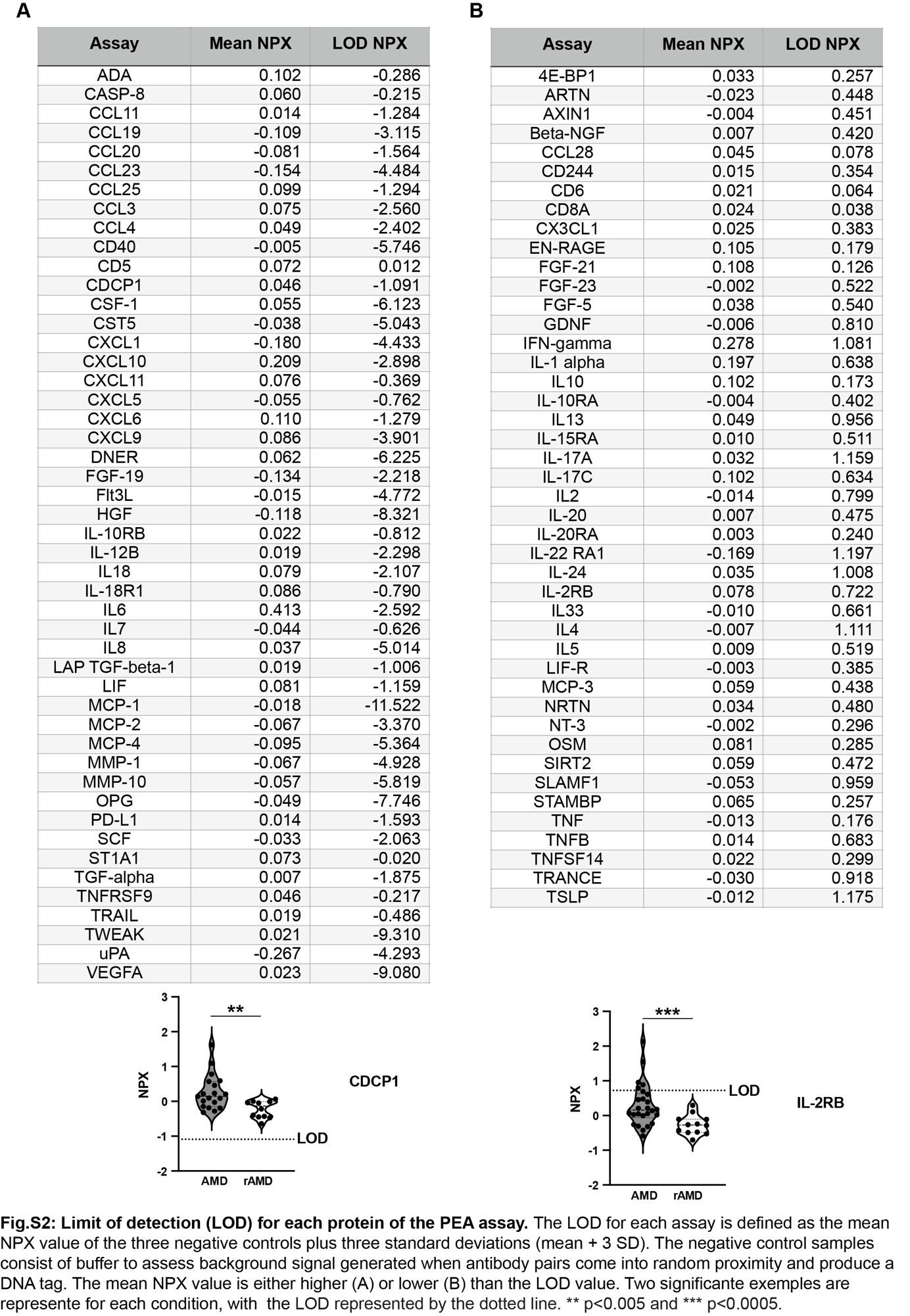

Supplement: Supplementary file 1 [file Supplementaryfile1.docx]
